# Culture-Independent Single-Cell PacBio Sequencing Reveals Epibiotic *Variovorax* and Nucleus Associated *Mycoplasma* in the Microbiome of the Marine Benthic Protist *Geleia* sp. YT (Ciliophora, Karyorelictea)

**DOI:** 10.3390/microorganisms11061500

**Published:** 2023-06-05

**Authors:** Xiaoxin Zhang, Luping Bi, Eleni Gentekaki, Jianmin Zhao, Pingping Shen, Qianqian Zhang

**Affiliations:** 1 School of Ocean, Yantai University, Yantai 264003, China; xiaoxin22211@163.com (X.Z.);; 2 Key Laboratory of Coastal Environmental Processes and Ecological Remediation, Yantai Institute of Coastal Zone Research, Chinese Academy of Sciences, Yantai 264003, Chinajmzhao@yic.ac.cn (J.Z.); 3 Muping Coastal Environment Research Station, Yantai Institute of Coastal Zone Research, Chinese Academy of Sciences, Yantai 264003, China; 4 School of Science, Mae Fah Luang University, Chiang Rai 57100, Thailand; gentekaki.ele@mfu.ac.th; 5 Gut Microbiome Research Group, Mae Fah Luang University, Chiang Rai 57100, Thailand

**Keywords:** single cell microbiomics, symbiosis, *Mycoplasma*, *Variovorax*, marine benthic ciliate, FISH

## Abstract

Microbes in marine sediments constitute up to five-sixths of the planet’s total biomass, but their diversity is little explored, especially for those forming associations with unicellular protists. Heterotrophic ciliates are among the most dominant and diversified marine benthic protists and comprise hotspot niches of bacterial colonization. To date, studies using culture-independent single-cell approaches to explore microbiomes of marine benthic ciliates in nature are almost absent, even for the most ubiquitous species. Here, we characterize the major bacterial groups associated with a representative marine benthic ciliate, *Geleia* sp. YT, collected directly from the coastal zone of Yantai, China. PacBio sequencing of the nearly full-length 16Sr RNA genes was performed on single cells of *Geleia*. Fluorescence in situ hybridization (FISH) analysis with genus-specific probes was further applied to locate the dominant bacterial groups. We identified a *Variovorax*-like bacterium as the major epibiotic symbiont residing in the kineties of the ciliate host. We provide evidence of a nucleus-associated bacterium related to the human pathogen *Mycoplasma*, which appeared prevalently in the local populations of *Geleia* sp. YT for 4 months. The most abundant bacterial taxa associated with *Geleia* sp. YT likely represent its core microbiome, hinting at the important roles of the ciliate-bacteria consortium in the marine benthos. Overall, this work has contributed to the knowledge of the diversity of life in the enigmatic marine benthic ciliate and its symbioses.

## 1. Introduction

Single-celled protistan species are found in almost every environment on Earth and have adapted to survive and/or thrive under hostile conditions [1]. During the course of evolution under these conditions, many protists have formed diverse associations with prokaryotes and/or eukaryotes (e.g., mutualism, commensalism, or parasitism). The most studied symbiotic associations have been reported in ciliates [2,3,4] but also in Rhizaria [5,6], haptophytes [7], and planktonic algae [8]. These close relationships contribute to ecosystem processes, such as nutrient and biogeochemical cycling [9,10]. In contemporary times, protists have emerged as a valuable source for discoveries of microorganisms with specialized physiological and metabolic characteristics [11]. Recently, the focus of studies centering on protist–bacteria associations has begun to shift from a single or a few microbial symbionts to entire prokaryotic communities, otherwise known as the microbiome. These communities include both stable (i.e., true symbionts) and temporary (i.e., food organisms) members [2,12,13,14]. To date, microbiome research has primarily centered on multicellular organisms, while the same topic has rarely been explored in unicellular protists.

Marine sediments comprise complex habitats, as they are characterized by large fluctuations of abiotic ecofactors [15]. The deepest sediment layers not only lack oxygen but, on occasion, contain significantly high concentrations of sulfide, which inhibits aerobic respiration [16,17]. Heterotrophic free-living ciliates inhabiting interstitial environments are among the most dominant and diversified protistan groups in the marine benthic microfauna and mesofauna [11], playing significant roles in the flow of matter and nutrients [18,19]. The adaptations of marine benthic ciliates are reflected in their specialized morphologies (e.g., vermiform shape) [20], diversity of anaerobic metabolism [21], and symbiotic relationships with prokaryotes [1]. The large size of interstitial ciliates provides niches for both ecto- and endosymbionts [1]. The ciliate cell offers a great variety of cellular compartments suitable for bacterial colonization, e.g., the surface membrane, cytoplasm, nuclear apparatus, mitochondria, and even the perinuclear space [22]. Associations of bacteria with ciliates have been predicted as prevalent in marine benthic environments [23], but comprehensive studies on symbioses have so far been limited to a few cultivable or occasionally abundant taxa, e.g., *Metopus* [21,24], *Euplotes* [13,25], and a few species of the class Karyorelictea [26,27,28]. Among these, the complex microbial consortia associated with benthic ciliates were examined in a geleiid and a *Kentrophorus* species [26,28], hinting at the important roles played by their microbiomes.

Nonetheless, information on symbiotic relationships (or microbiomes) of marine benthic ciliates remains largely unexplored, including the most common and pervasive species, e.g., *Geleia*, *Paraspathidium* and *Trachelocerca* [23]. Despite the huge diversity of marine ciliates of the seafloor and the interstitial environment [29,30], only a few species can be established as monoclonal cultures. The microbial inhabitants of the sub-seafloor have been estimated to constitute up to five-sixths of the planet’s total biomass and one-third of its living biomass [31], but their diversity is little explored. In recent years, marine sediments have been a prominent source of new microbe lineages in the tree of life [32]. These microbial communities are a primary source of prey and also symbionts for marine heterotrophic ciliates [33,34]. It is, therefore, reasonable to assume that a large number ciliate–prokaryote associations are yet undiscovered in the marine benthos.

Members of the genus *Geleia* (Karyorelictea, Geleiidae) are conspicuous and comprise the most prevalent ciliates encountered in marine sediments worldwide [30,35]. These organisms are huge in length (up to 1100 μm) and possess a vermiform shape to swim along the surface of sediment grains [30,35]. *Geleia* species typically inhabit sediment layers that are hypoxic or depleted of oxygen altogether [35,36], reaching high abundance (10^3^ individuals/mL) at the oxic/anoxic interface and in anoxic water columns rich in sulfur [35]. Microscopy studies on *Geleia* revealed the absence of conventional mitochondria, indicating anaerobic respiration [23]. Electron microscopy and epifluorescence revealed bacterial ectosymbionts on four examined species, reaching an abundance of 2000–10,000 bacteria per cell [23]. Due to the lack of molecular characterization in these studies, the composition and taxonomy of the epibiotic prokaryotes remain unknown. It is not clear whether the epibiotic prokaryote is the only symbiont or if there are additional stable and/or temporary members associated with *Geleia* species. Ciliates in natural environments have been hypothesized to host potentially pathogenic bacteria hence serving as their natural reservoirs [12]. Given the pervasive and dominant nature of *Geleia* in the marine benthos, examining the microbiome of this ciliate would not only contribute to the knowledge of symbiosis diversity and ecology in this habitat but would also provide insights into its potential role as a pathogen reservoir.

Recent advances in single-cell–omics have been instrumental in overcoming difficulties of cultivation and revealing in situ interactions between protists, including ciliates and their microbiomes in natural samples [12,13,14,25,37]. These pilot studies have uncovered distinct microbiomes of ciliates collected from marine and freshwater habitats [13,14]. Typically, these studies have made use of the HiSeq platform, which generates 300 bp fragments. The PacBio platform generates much longer fragments, which can cover the full length of the 16S rDNA sequences (with >0.99 accuracy under a Hifi mode), hence, allowing accurate molecular identification of the bacteria of interest. Obtaining full-length 16S rDNA sequences is conducive to designing bacterial-species-specific probes for fluorescence in situ hybridization (FISH) analysis, which can visualize the location of bacterial signals on/in the host cells.

In this study, we investigated the bacterial communities associated with an unidentified *Geleia* species (hereafter referred to as *Geleia* sp. YT) from the coastal zone of Yantai, China, using PacBio sequencing on single-cell amplicons. Ciliate individuals were collected directly from natural samples on different dates. Genus-specific probes were designed, and FISH experiments were performed to visualize the major bacterial OTUs on/in the ciliate host cells.

## 2. Materials and Methods

### 2.1. Sample Collection and Morphological Observations

Individual cells of *Geleia* sp. YT were collected in autumn 2021 and summer 2022 at two sites 10 km apart along the coastal zone of Yantai, China (37°28′50″ N, 121°27′41″ E). The two sites are designated hereafter as Site A (37°29′2.4″ N, 121°27′32″ E) and Site B (37°28′14″ N, 121°28′24″ E). Specimens were collected from the black sulfide-rich zone of the sediment, approximately 10–15 cm below the surface. Sediment was transported to the laboratory and examined using a stereomicroscope (VIHENT VHL745, Shanghai, China). Living cells of *Geleia* sp. YT were observed using the bright field (BF) mode with a light microscope (Olympus BX51, Tokyo, Japan) at 100–1000× magnification. Counts and measurements of the living cells were carried out at a magnification of 400×.

### 2.2. DNA Extraction, PCR Amplification, and PacBio Sequencing

Three individual *Geleia* sp. YT cells collected on different dates (2021-09, 2022-06 and 2022-07; collected from site A) were used for genomic DNA (gDNA) extraction. Cells were picked from the sediment with a glass micropipette and washed with in situ seawater filtered with a 0.2 µm millipore filter (Pall Laboratory, Port Washington, NY, USA) three times to remove environmental bacteria adhering to their surface. The ciliate cells were then starved in filtered seawater for 3–4 h, allowing the hosts to digest potential bacterial food in the food vacuoles. Each of the starved individuals was then transferred into 1.5 mL tubes with a small drop of water for DNA extraction. The REDExtract-N-Amp Tissue PCR Kit (Sigma, St. Louis, MO, USA) was used to extract genomic DNA from single cells, according to Gong et al. [38].

To amplify the 18S rRNA gene of the ciliate host, two successive rounds of PCR (semi-nested) were carried out using the primers EukA (5′-AACCTGGTTGATCCTGCCAGT-3′) and 5.8SR (5′-TACTGATATGCTTAAGTTCAGCGG-3′) for the first PCR reaction and 82F (5′-GAAACTGCGAATGGCTC-3′) and U1492R (5′-GGTTACCTTGTTACGACTT-3′) for the second PCR reaction [28,39]. Amplification reactions were performed using MegaFi Fidelity DNA Polymerase (abm, Vancouver, Canada) following the manufacturer’s protocol. The conditions of the first PCR were as follows: initial denaturation at 98 °C for 30 s; 35 cycles of denaturation at 98 °C for 10 s, annealing at 56 °C for 30 s, and elongation at 72 °C for 60 s); and a final elongation step at 72 °C for 120 s. The same protocol was also applied for the second PCR, except that 30 cycles were used instead of 35. The PCR products were purified using a TIANgel Midi Purification Kit (Tiangen Bio. Co., Shanghai, China) and then inserted into a pCE2 TA/Blunt-Zero vector (5 min TA/Blunt-Zero Cloning Kit, Vazyme, Nanjing, China) for cloning purposes using competent Fast-T1 cells (Vazyme, Nanjing, China). White colonies were randomly selected and screened by PCR with the universal primers M13-20 and M13-26 (Vazyme, Nanjing, China). The conditions of the PCR were as follows: initial denaturation at 94 °C for 3 min; 35 cycles of denaturation at 94 °C for 60 s, annealing at 55 °C for 60 s, and elongation at 72 °C for 60 s); and a final elongation step at 72 °C for 10 min. For each of the three *Geleia* sp. YT isolates, five positive colonies carrying the proper-length inserts were sent for Sanger sequencing (RuiBiotech sequencing facility, Qingdao, China).

For each isolate, the diversity of associated bacteria was examined using the PacBio Sequel II platform to amplify the nearly full length of the 16S rRNA gene. The primers used were 27F (5′-AGAGTTTGATCMTGGCTCAG-3′) [40] and 1492R (5′-GGTTACCTTGTTACGACTT-3′) [41]. The PCR protocol was as follows: an initial denaturation step at 98 °C for 30 s); 30 cycles of denaturation at 98 °C for 10 s, annealing at 51 °C for 30 s, and elongation at 72 °C for 60 s); and a final elongation step at 72 °C for 120 s. The resulting amplicons were purified and sequenced (Frasergen Co., Ltd., Wuhan, China). The Circular Consensus Sequencing (CCS) mode was used to produce reads with high resolution at the base level. More than 5000 reads were generated for each sample.

### 2.3. Sequence Analysis of PacBio Data

Raw PacBio sequences were processed as per our previous studies [28]. In brief, the raw data were pretreated using the Pacific Biosciences SMRT link v.8.0 Workflow (https://www.pacb.com/support/softwaredownloads; accessed on 23 July 2022). The generated subreads were converted to raw CCS with a strict threshold (minPasses ≥ 3, minPredictedAccuracy ≥ 0.99) using the CCS method [42]. Adapters were identified and trimmed from the raw CCS using Cutadapt V3.2 [43], with a maximum error rate of 0.1. Raw CCS shorter than 1300 bp were filtered by Usearch [44]. The resulting clean CCS included sequences with mixed orientations. The reverse sequences were aligned and reoriented using MAFFT [45]. Ambiguous bases in the two ends of the CCS were removed, inferring the PCR primers 27F and 1492R. CCS with ambiguous lengths (>2500 bp) were removed manually.

Abundance analysis and the primary classification of the 16S rRNA gene were performed according to Fu et al. [46]. In short, chimeras were identified and eliminated by the Vsearch program integrated with QIIME v.1.8.0 [47], combined with manual examination using blastN against an NCBI database. Singletons were deprecated using Mothur v.1.34.4 [48]. The resulting data were clustered into operational taxonomic units (OTU) with a 97% sequence similarity threshold using QIIME v.1.8.0 [47]. The representative sequences of each OTU were used as queries for blast searches and further aligned against the SILVA ribosomal RNA gene database (SILVA version 128) [49]. Accurate classification for the OTUs was inferred by both the SILVA annotation and the 16S rRNA phylogenetic analysis. Abundance analysis for the OTUs was further performed to determine the dominant bacterial taxa using the Pacman package [50] implemented in R (v.3.6.2). Terminology of bacteria followed Hördt et al. [51].

### 2.4. Phylogenetic Analysis

Phylogenetic analysis was performed for the 18S and 16S rRNA genes to further classify the ciliate host and the dominant bacterial OTUs. For the 18S rDNA phylogeny, a dataset was assembled using the sequences derived from the three isolates (2021-09, 2022-06, 2022-07) along with 37 sequences of karyorelictid ciliates spanning the diversity of the class and five sequences of the class Heterotrichea, which were used as the outgroup. Separate phylogenetic analyses were performed for the major associated bacterial genera. A total of 31 16S rRNA sequences from the family *Comamonadaceae*, 52 sequences from the genus *Mycoplasma* and phylum *Tenericutes*, 31 sequences from the order *Rhodospirillales* and *Hyphomicrobiales* (class *Alphaproteobacteria*), and 44 sequences from the class *Methylobacteriaceae* and the order *Rhizobiales* were included in the datasets, respectively. Selection of outgroups for the 16S rDNA analysis referred to the most recent phylogenetic studies on the corresponding taxa [51,52,53,54].

The assembled 16S and 18S rRNA datasets were aligned using SINA (v1.2.11) integrated into the SILVA Web server (https://www.arb-silva.de/aligner/; accessed on 25 July 2022) with default parameters [55]. The alignments were refined manually to remove poorly aligned sites using SeaView v4.0 [56]. The final matrix used for tree analysis of *Geleia* sp. YT comprised 1588 nucleotide positions. For the seven major genera, 1557–1600 nucleotide positions in the final matrixes were subjected to the analysis, respectively. Maximum likelihood (ML) and Bayesian inference (BI) were applied for the phylogenetic analyses. For all datasets, the GTR + I + Γ model was selected as the best model for both algorithms by jModelTest 2.1.7 [57] using the Akaike Information Criterion (AIC). The ML trees of the rRNA genes were constructed using RAxML V. 8 [58] with 100 random taxon additions to generate the starting trees. Bootstrap support was inferred from 1000 pseudo-replicate datasets. The Bayesian inference (BI) approach was implemented using MrBayes v.3.2.2 [59]. Markov chain Monte Carlo (MCMC) simulations were run for 5,000,000 generations (with the default temperature parameter and a sampling frequency of 0.01) with two parallel runs; each run had four simultaneous chains. The mean standard deviations of the split frequencies based on the last 75% of generations were all lower than 0.01 at the end of the analysis. The first 12,500 trees (corresponding to 25% of generations) were discarded as burn-in.

### 2.5. Fluorescence In Situ Hybridization (FISH) and Fluorescence Microscopy

Four oligonucleotide probes were newly designed to target the 16S rRNA of genera *Variovorax* and *Mycoplasma*, according to Gong et al. [60] with slight modifications. The new probes were named Vari-264, Vari-623, Myco-692, and Myco-910. The specificities of the probes were confirmed in silico against the SILVA SSU r138.1 database using Test Probe 3.0 (https://www.arb-silva.de/search/testprobe/; accessed on 7 September 2022). The antisense probe NON338 was used as a negative control for the hybridization protocol [61]. The probe BONE23A specific for betaproteobacteria [62] and EUB338 I/II/III targeting bacteria [63,64] were also used in our FISH analysis to facilitate the virtualization of OTUs-*Variovorax*. The “Cy3” fluorochrome was applied to all probes (EUB338 I/II/III, BONE23A, Vari-264/623, Myco-692/910, and ALF968). The primary sequence and specificity of the six probes are shown in Table 1.

The whole-cell hybridization procedure was carried out according to Oma et al. [65]. Briefly, starved ciliates were transferred to polylysine-treated adhesive slides and fixed with Bouin’s solution (50%, final concentration) for 3 h in darkness. The slides were then dried at room temperature. Prior to the FISH assay, the slides were washed three times in distilled water and then dehydrated in a stepwise fashion over an ethanol gradient concentration of 30%, 50%, 80%, and 100%. The formamide dosage used for each probe was predicted on the mathFISH web server (http://mathfish.cee.wisc.edu/formamide.html; accessed on 7 September 2022) [66]. Accordingly, a formamide concentration of 40% for the Myco-692 probe and a concentration of 30% for all other probes were applied to remove non-specific hybridizations as much as possible. A volume of 36 µL of hybridization buffer (containing 20 mM Tris-HCl at pH 8.0, 0.9 M NaCl, 0.01% SDS (sodium dodecyl sulfate) and 40%/30% formamide) was added to the slides along with 4 µL of each of the targeting fluorescent probes (5 ng/mL of final concentration). The slides were then incubated at 46 °C for 3 h. After hybridization, the slides were infused in a wash buffer (20 mM Tris-HCl at pH 8.0, 450 mM NaCl, and 0.01% SDS) for 15 min at 48 °C and then chilled using distilled water three times for 30 s. Antifade mounting medium (Beyotime, Nantong, China) and DAPI (50 ng/mL) were used to cover the specimens on the slides for 15 min in darkness. Slides were observed using an epifluorescence microscope (Olympus BX61, Olympus, Tokyo, Japan) equipped with a SPOT RT3 digital camera (SPOT Imaging Solutions, Sterling Heights, MI, USA) and mercury lamp accessories (Olympus U-RFL-T, Olympus, Tokyo, Japan). Image-pro plus 6.0 was used to color the photomicrographs.

## 3. Results

### 3.1. Morphology and Molecular Phylogeny of the Host Ciliate Geleia sp. YT

**Figure 1:**
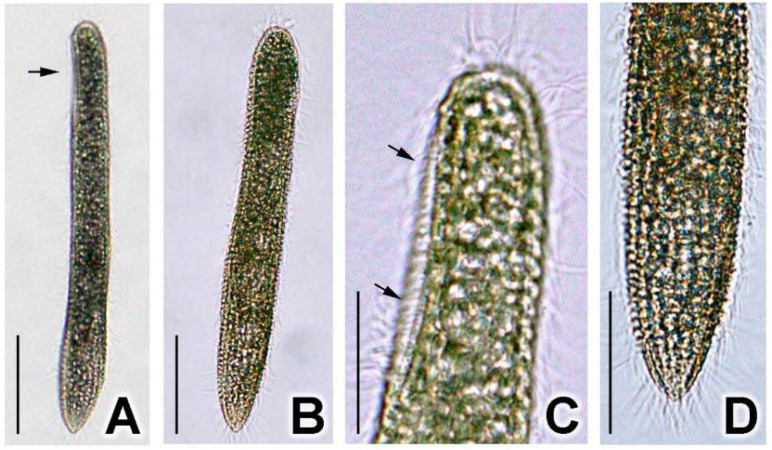
Photomicrographs of *Geleia* sp. YT from living cell with the light microscope. (**A**) Lateral view. Typical individual showing straight shape while gliding on the substrate. Buccal field is located at the anterior end (arrows); (**B**) Dorsal view. Typical individual showing straight shape while gliding on the substrate. (**C**) Detail of anterior end, lateral view. Arrow marks the buccal field. (**D**) Detail of posterior end, dorsal view. Scale bars = 25 μm (**A**,**B**); 10 μm (**C**,**D**).

Ciliate cell size in vivo was about 70–100 × 9–10 μm (n = 10, SD = 9.5 and SD = 0.6 for length and width, respectively). Cells were vermiform and cylindrical, with the posterior end slightly narrowed (Figure 1A,B). The body was flexible and slightly contractile (Figure 1A,B). The buccal field was 18–25 μm long, taking up about a quarter of the ciliate body (Figure 1A,C). The cell membrane was packed with inclusions of about 1–3 μm in diameter, rendering the cell slightly dark-brown at lower magnifications (Figure 1A,B). At higher magnifications, the cell was almost opaque (Figure 1C,D). Usually, two macronuclei and one micronucleus were observed (10/19 cells; n = 19).

**Figure 2:**
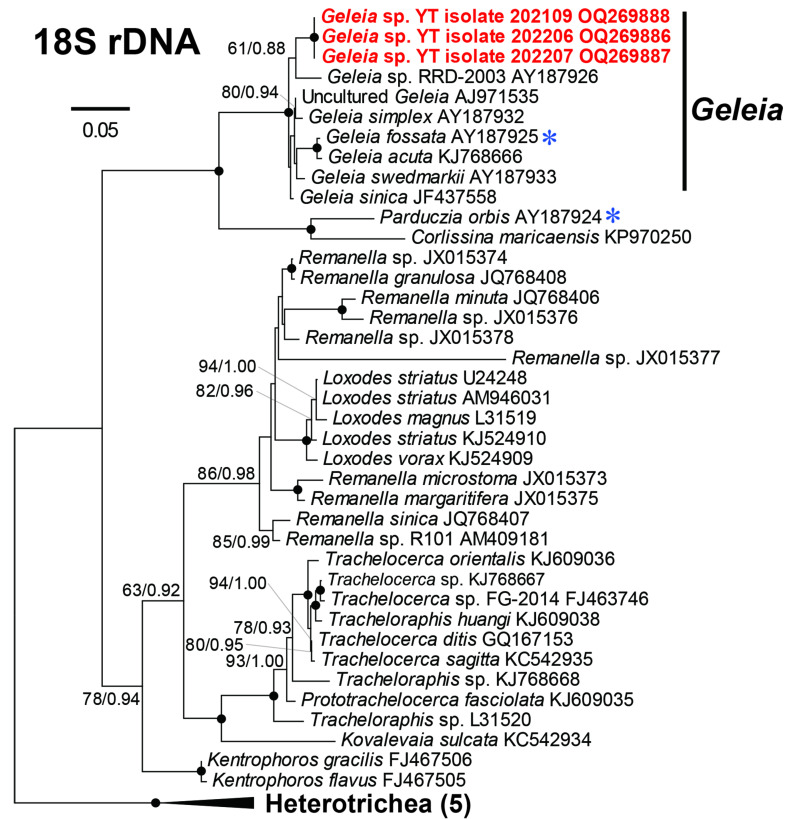
Maximum likelihood (ML) tree based on the 18S rRNA genes showing the positions of *Geleia* sp. YT (in red) with a GTR + I + Γ model. The numbers on the nodes represent the bootstrap values of the ML and the posterior probability of the Bayesian inference (BI). The black dot on the node represents the bootstrap value/posterior probability > 95%/0.95. Numbers with the bootstrap value <50% are not shown. The species marked with blue asterisks have been reported with symbionts. The sequences newly obtained in this study are highlighted in red bold. GenBank accession numbers are given following the species names. All branches are drawn to scale. The outgroup Heterotrichea comprised of species *Stentor roeseli* (AF357913), *Stentor amethystinus* (AY775566), *Eufolliculina uhligi* (U47620), *Blepharisma americanum* (M97909), and *Spirostomum ambiguum* (L31518).

The 18S rRNA sequences acquired from the three isolates of *Geleia* sp. YT (designated as isolates 2021-09, 2022-06, and 2022-07) were found to be identical both within the isolate (among clones) and across different isolates. One representative clone sequence from each isolate was used in the subsequent phylogenetic analysis. The sequences were deposited in GenBank under the accession numbers OQ269886–OQ269888. The acquired 18S rRNA gene of *Geleia* sp. YT was 1446 bp long and had a GC content of 50.90%. A blastN search against the NCBI database showed the sequence shared the highest identity (98.5%) with *Geleia sinica* (JF437558) but differed in 24 bases from the latter. In the phylogenetic trees, *Geleia* sp. YT fell into the monophyletic clade of genus *Geleia* with full support but showed no strong affiliations to the known species within the genus (Figure 2).

### 3.2. Diversity of Bacteria Associated with Geleia sp. YT

**Figure 3:**
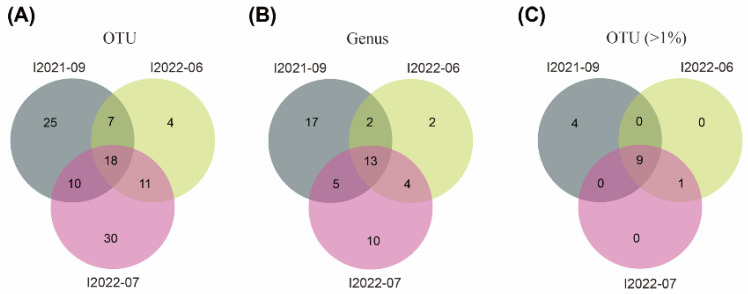
Venn diagrams showing numbers of OTUs (**A**), genus (**B**), and OTUs (relative abundance per individual > 1%) (**C**) shared by three individuals of *Geleia* sp. YT. I2021-09, I2022-06 and I2022-07 represent different individuals of *Geleia* sp. YT.

The clean data of CCS sequences derived from PacBio sequencing were deposited in GenBank with the bioproject number PRJNA924002. After the removal of chimeras and reads of low quality and ambiguous length (>2500 bp), a total of 2332, 2714, and 6372 high-quality reads for individuals I2021-09, I2022-06, and I2022-07, respectively, were clustered into 105 OTUs, corresponding to 56 potential genera. Among these, 18 OTUs belonging to 13 genera were commonly shared by the three individuals (Figure 3). When only considering measurable OTUs of >1% relative abundance per individual, the proportion of OTUs (9/14) shared by all three individuals increased (Figure 3).

**Figure 4:**
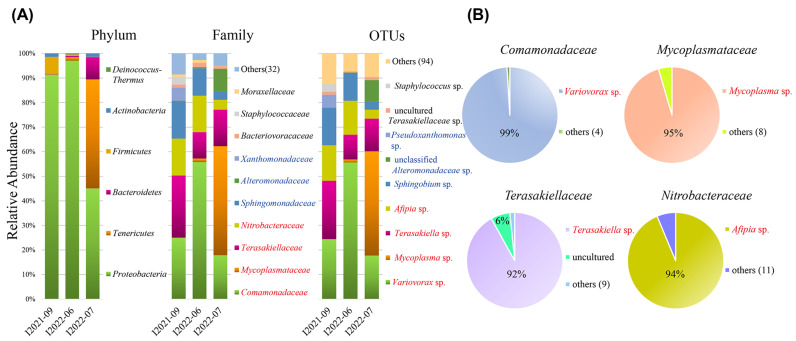
Relative abundances of the major bacterial taxa in *Geleia* sp. YT (**A**) and OTUs comprising the top four abundant families in the total reads (**B**), inferred by the PacBio data. Names of the top 4 abundant families, genera, and OTUs are highlighted in red. Medium families and genera (with OTUs > 5% per individual) are highlighted in blue. I2021-09, I2022-06, and I2022-07 represent different individuals of *Geleia* sp. YT.

Based on the total reads, the phylum *Proteobacteria* accounted for a considerable proportion of the OTUs (69.09%), followed by *Tenericutes* (23.18%) and *Bacteroidetes* (4.86%) (Figure 4A). The top four most abundant bacterial families comprised the majority of the total detected bacterial diversity (78%; Figure 4A). These were *Comamonadaceae* (29.43%), *Mycoplasmataceae* (23.18%), *Terasakiellaceae* (16.12%), and *Nitrobacteraceae* (formerly *Bradyrhizobiaceae*) (9.31%) (Figure 4A). Of the total reads of the three individuals, the four families were overwhelmingly predominated by singlet OTUs, namely the OTU *Variovorax* sp. (99%), OTU *Mycoplasma* sp. (95%), OTU *Terasakiella* sp. (92%), and OTU *Afipia* sp. (94%) (Figure 4B). Most of the four dominant OTUs were comparably detected in all three individuals (Figure 4A, Table 2). One exception was the OTU *Mycoplasma* sp., which was found solely in the isolate I2022-07 (42.5% per individual) but was merely detectable in the other two individuals (0.09% and 1.2% per individual; Table 2).

We also identified several families with medium abundances (1–8% of the total reads) in the PacBio data. These included *Sphingomonadaceae* (8.1%), *Alteromonadaceae* (4.9%), and *Xanthomonadaceae* (1.2%) (Figure 4A). All of these families were represented by singlet OTUs, classified as *Sphingobium* sp. unclassified *Alteromonadaceae* and *Pseudoxanthomonas* sp., respectively. Of the different ciliate individuals, the relative abundances of these OTU varied obviously (Figure 4A; Table 2), i.e., *Sphingobium* sp. (15.2% vs. 11.0% vs. 3.4%), *Pseudoxanthomonas* sp. (0.2% vs. 0.5% vs. 8.6%), and *Alteromonadaceae* sp. (5.1% vs. 0% vs. 0%).

An additional two OTUs of measurable but low abundance (>1% relative abundance in either individual) were identified, e.g., an uncultured *Terasakiellaceae sp.* (1.04% of total reads) and *Staphylococcus* sp. (0.6% of the total reads) (Figure 4A). Because the relative abundances of these OTUs in an individual were all lower than 5%, we did not consider them as potential symbionts in subsequent analyses [13].

### 3.3. Phylogenetic Analyses of the Abundant Bacterial OTUs Associated with Geleia sp. YT

**Figure 5:**
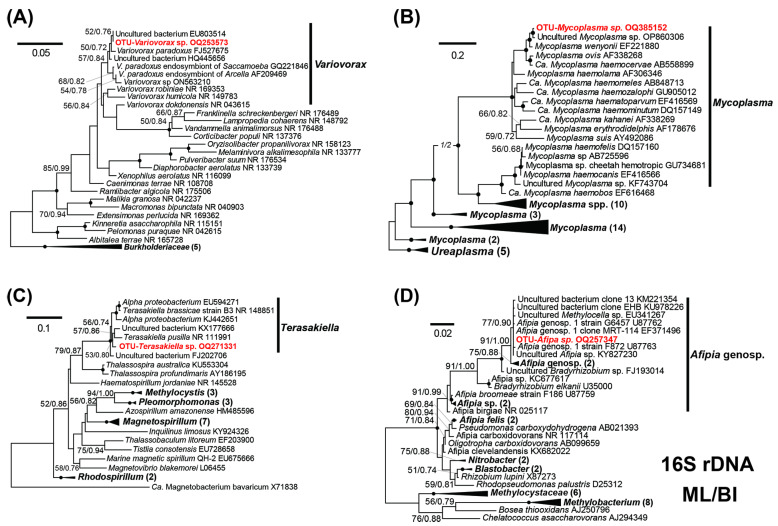
Maximum likelihood (ML) trees based on the 16S rRNA genes, showing phylogenetic positions of OTUs in the top 4 abundant genera (in red): *Variovorax* sp. (**A**), *Mycoplasma* sp. (**B**), *Afipia* sp. (**C**), and *Terasakiella* sp. (**D**). All trees were constructed under a GTR + I + Γ model. The numbers on the nodes represent the bootstrap values of ML and the posterior probability of the Bayesian inference (BI). The black dot on the node represents the bootstrap value/posterior probability >95%/0.95. Numbers with bootstrap values <50% are not shown. The sequences newly obtained in this study are highlighted in red bold. Genbank accession numbers are given following the species names. All branches are drawn to scale.

For the seven high/medium-abundant OTUs with an individual abundance higher than 5% (Table 2), phylogenies were inferred using the full length of the 16S rRNA gene to curate the initial classifications with blastN against the SILVA database. For brevity purposes, only the phylogenetic trees of the four most dominant genera are shown in Figure 5, while the rest are shown in Figures S1–S3.

In the phylogenetic trees, the OTU *Variovorax* sp. fell into the clade of the genus *Variovorax*, clustering with other *V. paradoxus* strains (Figure 5A). The sequence of the OTU *Variovorax* sp. was highly similar to an environmental sequence (EU803514) and a *Variovorax paradoxus* (FJ527675) representing a symbiotic bacterial strain isolated from a root nodule of a legume host (Table 2). The 16S rRNA gene of OTU *Mycoplasma* sp. was affiliated with other *Mycoplasma* sequences in the phylum *Tenericutes* (Figure 5B). The OTU robustly clustered with an uncultured *Mycoplasma* species (OP860306) and the species *M. wenyonii* (Figure 5B), differing by 3 and 5 bases, respectively. The OTU *Terasakiella* sp. and OTU *Afipia* sp. fell into monophyletic clades of the genera *Terasakiella* and *Afipia* (Figure 5C,D). The OTU *Terasakiella* sp. did not show a close affiliation to any known species; its 16S rDNA sequence is most similar to an uncultured bacterium collected in a sea oil reservoir (EU594271), but the two differ by 32 bases (Table 2). The OTU *Afipia* sp. fell into a clade comprised of mainly unnamed genospecies, which were divergent from the named species (Figure 5D).

Classifications for the three medium-abundant OTUs, namely *Sphingobium* sp., *Alteromonadaceae* sp., and *Pseudoxanthomonas* sp., were also curated by trees (Figures S1–S3). The classification of the OTU *Alteromonadaceae* sp. was amended according to the trees, which was misclassified initially with blastN against the SILVA database as a *Fluviicola* (Table 2).

### 3.4. Fluorescence In Situ Hybridization (FISH)

For the top 2 abundant OTUs in the total reads, namely the OTU *Variovorax* sp. and OTU *Mycoplasma* sp., the specific probes Vari-264/Vari-623 and Myco-692/Myco-910 were newly designed for FISH analysis. In silico analysis of the Vari-264/Vari-623 probes showed high specificity for the family *Comamonadaceae* in the SILVA SSU r138.1 database (accessed on 7 September 2022). The probe Vari-623 precisely matched the family *Comamonadaceae* (110/110) in the database, while the Vari-264 probe matched a smaller range of *Comamonadaceae* (228/294). Being in silico hybridized against the 105 OTUs obtained in this study, Vari-623 and Vari-264 only matched the OTU *Variovorax* sp. and another less abundant *Variovorax*-like OTU. The two new probes of OTU *Mycoplasma* sp. (Myco-692 and Myco-910) matched the genus *Mycoplasma* precisely (34/34 and 53/53) in both the SILVA database and our PacBio dataset.

**Figure 6:**
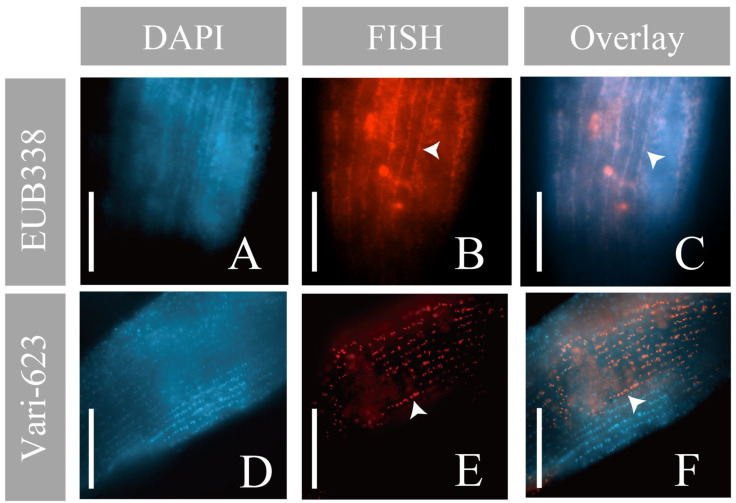
Micrographs of *Geleia* sp. YT with OTU *Variovorax* sp. (**A**,**D**) DAPI staining; (**B**,**E**) fluorescence in situ hybridization (FISH) using EUB338 (**B**) and Vari-623 probes (**E**); (**C**,**F**) overlay of the DAPI and FISH results. Signals were placed along the kineties (arrowheads). Scale bars = 10 μm (**A**–**F**). The two cells hybridized with EUB338 and Vari-623 were collected from site A.

To visualize the location of OTU *Variovorax* sp. on/in *Geleia* sp. YT, DAPI and the probes were applied separately to a total of 12 cells of *Geleia* sp. YT (2 cells with EUB338, 2 with BONE23A, 6 with Vari-623, and 2 with Vari-264) sampled on seven different dates (09/2021 and 06/2022–10/2022). DAPI staining and hybridization with group-specific (EUB338 and BONE23A) and OTU-specific probes (Vari-623/264) consistently revealed positive signals along the ciliated grooves, which were linearly arranged on the cell surface (Figure 6, Figures S4 and S5). These linearly arranged signals were uniformly distributed over the entire length of the cell body (Figures S4E–H and S5A–D). Signals from the *Variovorax*-specific probe (Vari-623) seemed to appear exclusively on the surface, and rod-shaped bodies were distinguishable in some cells (Figure 6E). The size and morphology of the *Variovorax* signals were quite similar to the DAPI-stained bodies (Figure 6D,F) and both were placed along the kineties (Figure 6F).

**Figure 7:**
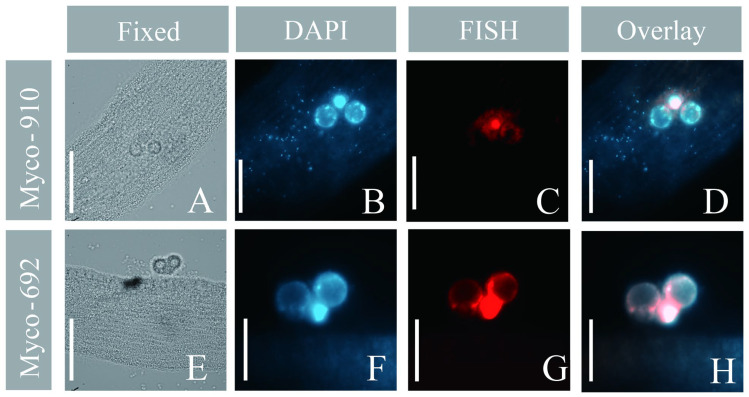
Micrographs of *Geleia* sp. YT with OTU *Mycoplasma* sp. (**A**–**H**). (**A**,**E**) Microphotographs of the fixed cells in the bright field; (**B**,**F**) DAPI staining; (**C**,**G**) fluorescence in situ hybridization (FISH) using Myco-910 (**C**) and Myco-692 (**G**); (**D**,**H**) overlay of the DAPI and FISH results. Scale bars = 10 μm (**A**,**E**); 5 μm (**B**–**D**,**F**–**H**). The two cells hybridized with Myco-910 and Myco-692 were collected from site A and site B, respectively.

The *Mycoplasma* probes (Myco-692/910) were applied to a total of seven *Geleia* sp. YT cells (3 with Myco-692 and 4 with Myco-910) that were collected on seven dates covering each month between 07/2022 and 10/2022. Similar results were observed in all the examined specimens. DAPI-stained bodies were shown in (or around) the nucleus and scattered on (or in) the somatic area (Figure 7B and Figure S6), while positive signals of *Mycoplasma* were observed only in the nuclear area. Both the DAPI bodies and the OTU *Mycoplasma* signals were intensive and concentrated near/around the nuclear area (Figure 7B,C,F,G and Figure S6). In most cells we examined, fluorescence signals from the DAPI and the OTU *Mycoplasma* probes aligned well (Figure 7F–H and Figure S6).

The common *alphaproteobacteria*-specific probe (ALF968) was applied to visualize another three OTUs with high/medium abundances in the total reads, namely the OTU *Terasakiella* sp., OTU *Apifia* sp., and OTU *Sphingobium* sp. Unfortunately, due to *Geleia* sp. YT populations disappearing in the natural samples after November 2022, we were not able to test the probe on cells of *Geleia* sp. YT. Alternatively, we applied the probe ALF968 on two cells of another *Geleia* species, *Geleia* sp. 2022-11, collected at the same sites in Yantai. The result showed that signals corresponding to *Alphaproteobacteria* were present mainly in the cytoplasm of *Geleia* sp. 2022-11 (Figure S4K,L) and were clearly distinguishable from the DAPI-stained bodies of the epibiotic bacteria on the cell surface (Figure S4J,L).

## 4. Discussion

### 4.1. The Newly Collected Ciliate Geleia sp. YT

At present, 18 species of *Geleia* have been described. Among these, molecular data (18S rRNA gene) are available for only five, namely *G. acuta*, *G. fossata*, *G simplex*, *G. sinica*, and *G. swedmarkii*. Epibiotic bacteria have been recorded on three species, namely *G. fossata*, *G. murmanica,* and *G. vacuolata* [23]. In the phylogenetic trees, the newly obtained 18S rRNA gene of *Geleia* sp. YT did not show a strong affiliation to any other known species of the genus (Figure 2). Therefore, we refrained from arranging *Geleia* sp. YT to any known *Geleia* species.

### 4.2. Consistent Presence of Epibiotic Variovorax-like Bacteria and Their Potential Roles

As seen from the PacBio results, the OTU *Variovorax* sp. was the most abundant in the total reads (29.15%, corresponding to app. 2939 CCS) (Figure 4A). The FISH experiments with *Variovorax*-specific and group-specific probes revealed its location on the cell surface of the host (Figure 6). FISH signals were observed consistently on 12 host cells that were sampled on seven different dates, indicating that these bacteria are “true”, stable epibionts of *Geleia* sp. YT, rather than environmental contaminants. Earlier studies using SEM/TEM and epifluorescence also observed unidentified epibiotic bacteria on four *Geleia* species [23]. The epifluorescence signals recovered on *Geleia* sp. YT are similar to those recorded on other *Geleia* species (*G. fossata* in particular) in terms of their surface location, rod shape, bacterial biomass, and their precise arrangement along the host dikinetids. Nevertheless, it is immature to deduce that the epibiotic bacteria on the other *Geleia* species are also *Variovorax*-like bacteria. Future studies focusing on molecular characterization of additional *Geleia* species epibionts would be informative in this regard.

*Variovorax* species have been found ubiquitously in the rhizosphere as plant symbionts [67]. Free-living strains have been mainly recorded in soil [68,69] and freshwater environments [70,71], with very few strains isolated from deep marine sediments [72]. Symbioses of *V. paradoxus* with unicellular protists have been only reported in freshwater amoebae as endosymbionts of *Saccamoeba* [73], *Arcella* [74], and *Platyamoeba* [75]. The results of our work extend the range of *Variovorax* hosts by adding a new niche, the cell surface of a marine benthic ciliate.

*Variovorax* species are versatile chemoorganotrophs/lithoautotrophs capable of utilizing a wide range of inorganic or organic compounds. Many species participate in the degradation of both recalcitrant biogenic and anthropogenic compounds, including sulfur and amino acid pollutants, pesticides, and S-metabolites [76]. The ability to produce beneficial metabolites and consume harmful chemicals, e.g., toxic contaminants, makes *Variovorax* species beneficial symbionts of plants and nitrogen-fixing microbes [67]. Accordingly, the epibiotic *Variovorax* sp. on the benthic/interstitial ciliates may play similar roles, e.g., providing protection or nutrition. Indeed, nutrition-based symbioses between ciliates and epibiotic prokaryotes have been documented [1,77,78]. Nevertheless, the nature of the relationship between the *Geleia* sp. YT and the epibiotic *Variovorax* sp.remains to be elucidated.

### 4.3. Intranuclear Mycoplasma-like Bacteria of Geleia sp. YT

*Mycoplasma* was the most abundant OTU in the individual I2022-07 (42.5%), but it was merely detectable in the other two cells (0.09% and 1.2%) (Figure 4A). In the seven specimens sampled monthly between 07/2022 and 10/2022, our FISH experiment revealed repeatedly strong *Mycoplasma* signals in the hosts (Figure 7A–H and Figure S6A–C), implying that *Mycoplasma* seems to be a rather prevalent symbiont in the local populations of *Geleia* sp. YT during 07/2022–10/2022.

Members of *Mycoplasma* comprise well-known pathogens of humans [79,80] and a wide array of vertebrates [81]. However, associations of *Mycoplasma* with unicellular protists have been rarely recorded, except for the symbiosis of *M*. *hominis* with the human parasite *Trichomonas vaginalis* [82]. Remarkably in this study, *Mycoplasma* was found to be specifically associated with the nucleus of *Geleia* sp. YT (Figure 6A–H). Intranuclear *Mycoplasma* in eukaryotes has been documented only seldom [83], with no record in ciliates. The most well-known intranuclear bacteria in ciliates have mainly referred to *Holospora* and *Holospora*-like taxa, which are found in the micro- and macronuclei of *Paramecium* species [84,85]. *Mycoplasma* is a very well-studied human pathogen; however, its pathogenesis does not involve nuclear localization [86], with one exception [87]. Hence, the association of *Mycoplasma* with the ciliate nucleus and their prevalent occurrence in the population (found in cells collected in 4 months) might represent a novel relationship of the pathogen with eukaryotes.

The depletion of DAPI-stained bodies near the nuclei (Figure 7B,F) suggests low DNA content of the host cell, hinting at the parasitic potential of the *Mycoplasma* sp. in *Geleia* sp. YT. Since *Mycoplasma* bacteria have small genomes and lack many pathways necessary for survival, they rely highly on a host cell for their metabolic needs [88,89]. Hence, the bacteria may gain nuclear fractions from the host for replication, as is the case with other intranuclear bacterial lineages [90,91]. The ability of *Mycoplasma* strains to replicate from nuclear fractions of the host has been documented [92]. Another explanation for the solely nuclear location of *Mycoplasma* in the ciliate cell could be protection from cytoplasmic processes of the host, such as autophagy [83].

In this work, the host list of *Mycoplasma* is extended to include the free-living ciliate *Geleia*, which is cosmopolitan and dominant in the marine benthic ecosystem. This is the first record of *Mycoplasma*-like bacteria infecting common heterotrophic micro-eukaryotes in nature, which has implications for studies on microbial transmission in marine benthic micro-communities.

### 4.4. Microbiomes of the Marine Benthic Ciliate Geleia sp. YT

In this work, the microbiome profiles of three individual ciliate cells of the same species were explored using PacBio CCS to obtain nearly complete 16S rRNA sequences. Only a few studies have explored the microbiomes of single ciliate cells [12,25], and even fewer have dealt with marine benthic ciliates [26,28]. At the phylum level, *Proteobacteria* dominated in two microbiomes (i.e., I2021-09 and I2022-06) and were nearly equally abundant with *Tenericutes* in the one infected by *Mycoplasma* (I2022-07, Figure 4A). The dominance of *Proteobacteria* was also noted in the microbiome of the karyorelictid ciliate *Kentrophoros*, which was collected on the chemocline layer at the same sites as *Geleia* [28]. Intriguingly, the one geleiid (I2021-09) infected with *Mycoplasma* also had a notable abundance of *Bacteroidetes* (Figure 4A).

At the OTU level, aside from *Variovorax* sp. and *Mycoplasma* sp., the OTUs with abundances higher than 5% per individual, i.e., *Afipia* sp., *Terasakiella* sp., *Sphingobium* sp., and an unidentified *Alteromonadaceae*, accounted for app. 90% of the overall relative abundance of the three microbiomes (Table 2). In the individual cells, the top four OTUs (*Variovorax* sp., *Terasakiella* sp., *Sphingobium* sp., and *Afipia* sp.) were the same in the two uninfected cells and in the infected cell when the parasite *Mycoplasma* was excluded. These might represent bacteria preferentially associated with the ciliate *Geleia* sp. YT.

Among these, members of *Afipia* are opportunistic pathogens in human skin or blood [93,94] and are resistant to phagocytosis [95], indicating their symbiont potential. Free-living *Afipia* species play biogeochemical roles as degraders of environmental recalcitrant C1-sulfur compounds [96,97]. *Terasakiella* and *Sphingobium* have been commonly isolated in soil, intertidal zones [98], and aquatic environments [54,97]. Both genera are chemolitotrophs [99,100]. *Sphingobium*, most especially, is capable of degrading a variety of chemicals in the environment, with the potential to also degrade sulfur-containing compounds [101]. These information hints at important roles of the ciliate-bacteria consortium in the marine benthos. Nevertheless, the role of the microbiome of *Geleia* sp. YT remains to be verified by evidence from metabolomics and physiologic studies [102].

Moreover, comparing the microbiomes of *Kentrophoros* sp. YE from the same location and *Geleia* sp. YT, none of the major bacteria are shared beyond the phylum level. Therefore, we suppose that the core microbiome might be species/genus-specific and closely associated with the environmental conditions at the time of sampling, as are those revealed in single-cell cases of other ciliates, e.g., *Euplotes*, *Stentor* and *Paramecium* [12,13,14].

## 5. Conclusions

In this work, we investigated the ciliate species *Geleia* sp. YT, which represents one of the most pervasive heterotrophic protists in marine benthic habitats, and explored the diversity of its microbiome using culture-independent single-cell PacBio sequencing. We provided FISH evidence of an epibiotic bacteria along the dikinetids of the host ciliate. We identified and classified this bacterium as *Variovorux* sp. A potential parasitism relating to the human pathogen *Mycoplasma* was also detected and associated with the nuclei. The association with the ciliate nucleus and the prevalent occurrence of the microbe in the ciliate cells collected during 4 months has implications for the epidemiology of the pathogen. The four most abundant taxa are suggested as core microbiomes preferentially associated with the ciliate *Geleia* sp. YT. Future studies using single-cell omics should be further carried out on *Geleia* to verify the functions of the microbiome in the host. Microbiomes of a wider breadth of marine ciliates should also be performed to elucidate additional protist–bacteria associations from the marine benthos. These avenues of investigation will expand our knowledge of the diversity of life in the enigmatic marine benthic environment and the potential impacts of these microbial populations on major biogeochemical cycles.

## Figures and Tables

**Table 1:** Oligonucleotide probes used in FISH for the detection of the OTU-no1-4 bacteria.

Probe	Sequence (5′–3′)	Specificity	Data Source
Vari-264	TTCGATCTGTAGCTGGTC	*Comamonadaceae*	Present study
Vari-623	CTGTGACTGCATCGCTGG	*Comamonadaceae*	Present study
Myco-692	TGAGGAACACCAGAGGCT	*Mycoplasma*	Present study
Myco-910	TGAACAAGTGGTGGAGCA	*Mycoplasma*	Present study
BONE23A	GAATTCCACCCCCCTCT	*Betaproteobacteria*	[62]
ALF968	GGTAAGGTTCTGCGCGTT	Mainly *alphaproteobacteria*	[63]

**Table 2:** The most common OTUs (relative abundance >5% per individual) retrieved from *Geleia* sp. YT microbiomes.

OTU Family	OTU Name	OTU Abundance	Sequence Length	GC Content (%)	Matched Sequences (Genbank Accession Number) and Identity	Matched Known Species (Genbank Accession Number) and Identity	Curated Classification	Ecological Category
		Total/I2021-09/I2022-06/I2022-07 (%)						
*Comamonadaceae*	OTU *Variovorax* sp.	29.2/24.3/55.7/17.7	1479	54.70	Uncultured bacterium (EU803514) 100%	*Variovorax paradoxus* (FJ527675) 100%	*Variovorax* sp.	Symbiosis/free-living
*Mycoplasmataceae*	OTU *Mycoplasma* sp.	22.1/0.09/1.2/42.5	1435	44.60	Uncultured *Mycoplasma* sp. (OP860306) 99.79%	*Mycoplasma wenyonii* (EF221880) 99.65%	*Mycoplasma* sp.	Parasitism/symbiosis
*Terasakiellaceae*	OTU *Terasakiella* sp.	14.8/23.8/10.2/13.3	1441	52.88	Uncultured bacterium from environmental samples (EU594271) 97.78%	*Terasakiella brassicae* (NR148851) 97.78%	*Terasakiella* sp.	Free-living
*Nitrobacteraceae*	OTU *Afipia* sp.	8.7/14.5/13.8/3.7	1435	55.47	*Afipia* genosp. (EF371496) 100%	*Afipia* genosp. (EF371496) 100%	*Afipia* sp.	Potential pathogens/ free-living
*Sphingomonadaceae*	OTU *Sphingobium* sp.	8.1/15.2/11.0/3.4	1431	54.65	*Sphingobium* sp. (AY689029) 100%	*Sphingobium limneticum* (AY689029) 99.86%	*Sphingobium* sp.	Free-living
*Alteromonadaceae*	OTU unclassified *Alteromonadaceae* sp.	4.6/0.2/0.5/8.6	1466	52.18	Uncultured bacterium from environmental samples (EU617867) 96.4%	*Fluviicola taffensis* (NR074547) 94.90%	unclassified *Alteromonadaceae* sp.	Free-living
*Xanthomonadiceae*	OTU *Pseudoxanthomonas* sp.	1.2/5.1/0/0	1491	55.33	*Pseudoxanthomonas mexicana* (CP095186) 100%	*Pseudoxanthomonas mexicana* (CP095186) 100%	*Pseudoxanthomonas* sp.	Free-living/symbiosis

## Data Availability

The datasets presented in this study can be found in online repositories. The names of the repository/repositories and accession number(s) can be found below: NCBI (accession numbers OQ269888, OQ269887, OQ269886, OQ253573, OQ271331, OQ257347, OQ385152, and PRJNA924002).

## Supplementary Materials

The following supporting information can be downloaded at: https://www.mdpi.com/article/10.3390/microorganisms11061500/s1, Figure S1: Maximum likelihood (ML) tree based on the 16S rRNA genes showing the positions of OTU *Sphingobium* (in red) with a GTR + I + Γ model; Figure S2: Maximum likelihood (ML) tree based on the 16S rRNA genes showing the positions of OTU *Alteromonadaceae* sp. (in red) with a GTR + I + Γ model; Figure S3: Maximum likelihood (ML) tree based on the 16S rRNA genes showing the positions of OTU *Pseudoxanthomonas* sp. (in red) with a GTR + I + Γ model; Figure S4: Micrographs of *Geleia* sp. YT with universal probes; Figure S5: Micrographs of *Geleia* sp. YT with specific probes of OTU *Variovorax* sp.; Figure S6: FISHs with Myco-692/910 probes for *Geleia* sp. YT on different sample dates.
