# Supplementary material for: Culture-Independent Single-Cell PacBio Sequencing Reveals Epibiotic Variovorax and Nucleus Associated Mycoplasma in the Microbiome of the Marine Benthic Protist Geleia sp. YT (Ciliophora, Karyorelictea)
Source: Microorganisms. 2023 Jun 5;11(6):1500. doi: 10.3390/microorganisms11061500 (PMC10300761; doi:10.3390/microorganisms11061500)
Supplement: Supplementary file 1 [file microorganisms-11-01500-s001.zip › microorganisms-2389927-supplementary.pdf]

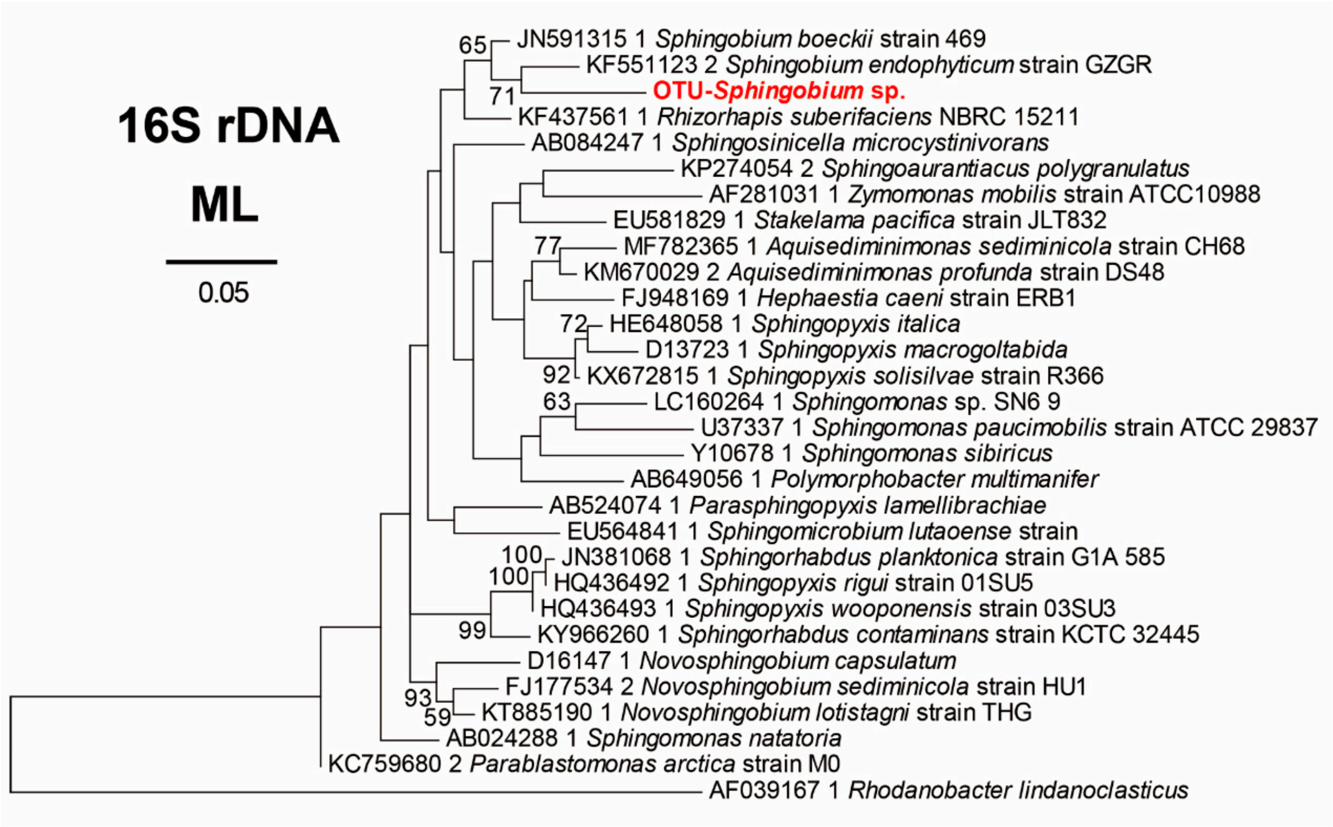

Figure S1 Maximum likelihood (ML) tree based on the 16S rRNA genes showing the positions of OTU-*Sphingobium* (in red bold) with a GTR + I + Γ model. The numbers on the nodes represent the bootstrap values of ML. Bootstrap values above 50% are represented. All branches are drawn to scale.

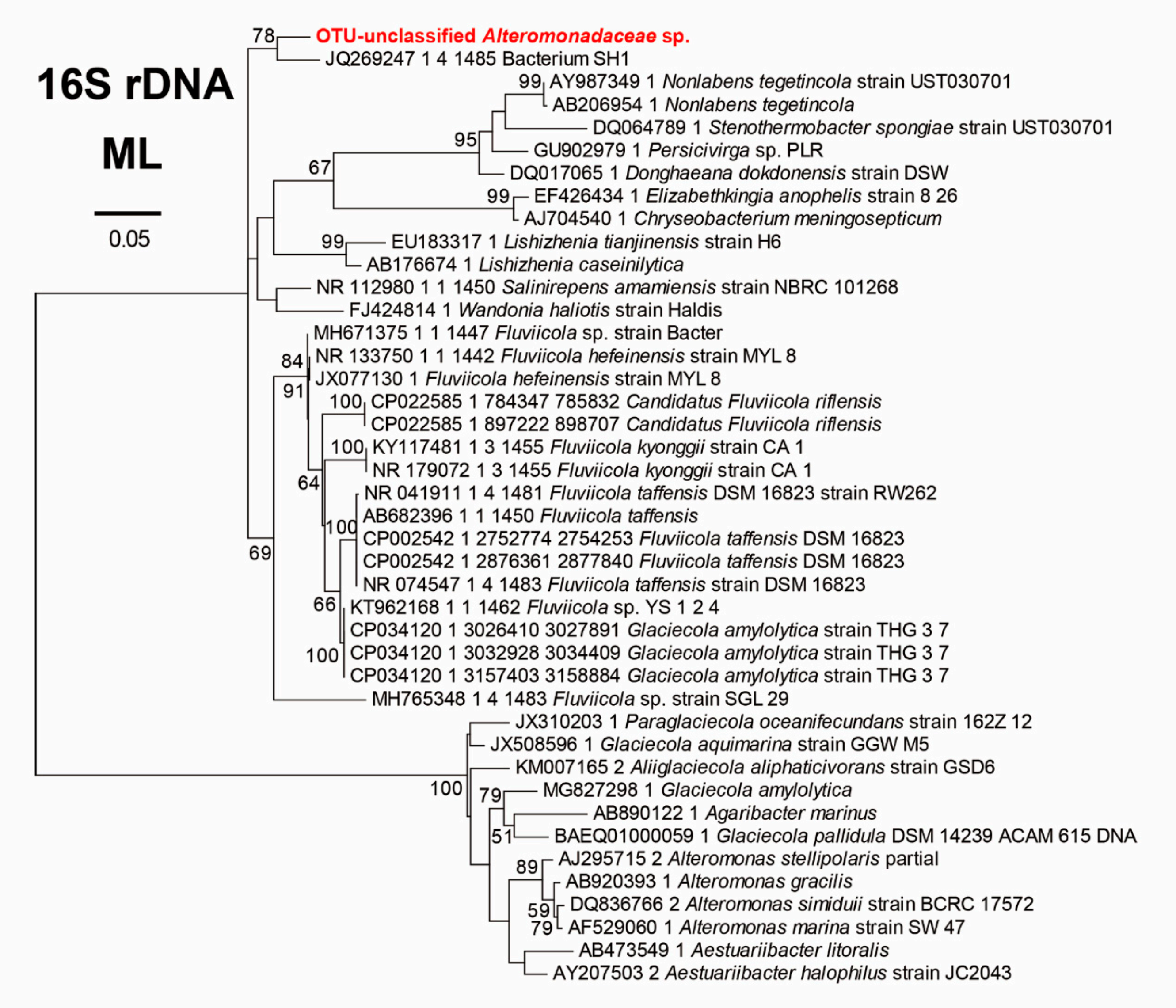

Figure S2 Maximum likelihood (ML) tree based on the 16S rRNA genes showing the positions of OTU-*Alteromonadaceae* sp. (in red bold) with a GTR + I + Γ model. The numbers on the nodes represent the bootstrap values of ML. Bootstrap values above 50% are represented. All branches are drawn to scale.

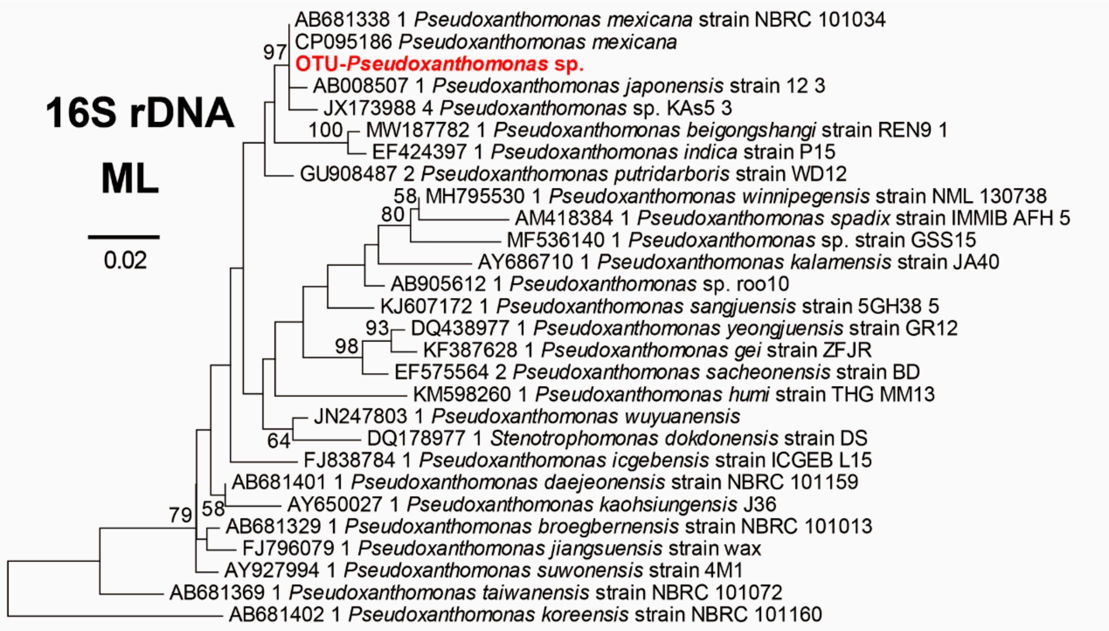

Figure S3 Maximum likelihood (ML) tree based on the 16S rRNA genes showing the positions of OTU-*Pseudoxanthomonas* sp. (in red bold) with a GTR + I + Γ model. The numbers on the nodes represent the bootstrap values of ML. Bootstrap values above 50% are represented. All branches are drawn to scale.

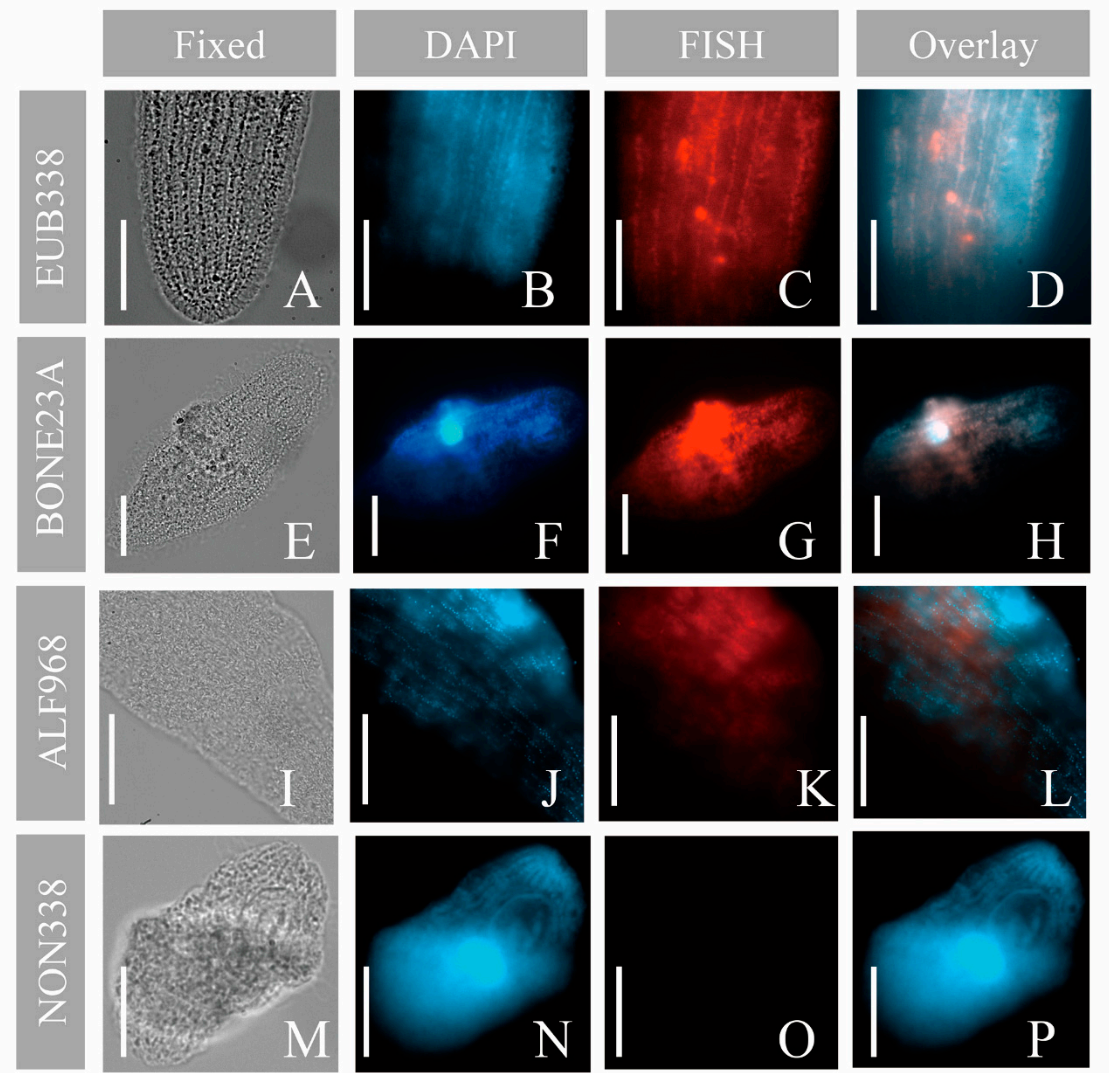

Figure S4 Micrographs of *Geleia* sp. YT with universal probes. (A, E, I, M) Microphotographs of the fixed cells in the bright field; (B, F, J, N) DAPI staining; (C, G, K, O), and fluorescence in situ hybridization (FISH) using EUB338 (C), BONE23A (G), ALF968 (K) and NON338 probes (O); (D, H, L, P) overlay of the DAPI and FISH. Scale bars = 10 μm (A–P). The cells EUB338, BONE23A and NON338 were collected from site A while the cell ALF968 was collected from site B.

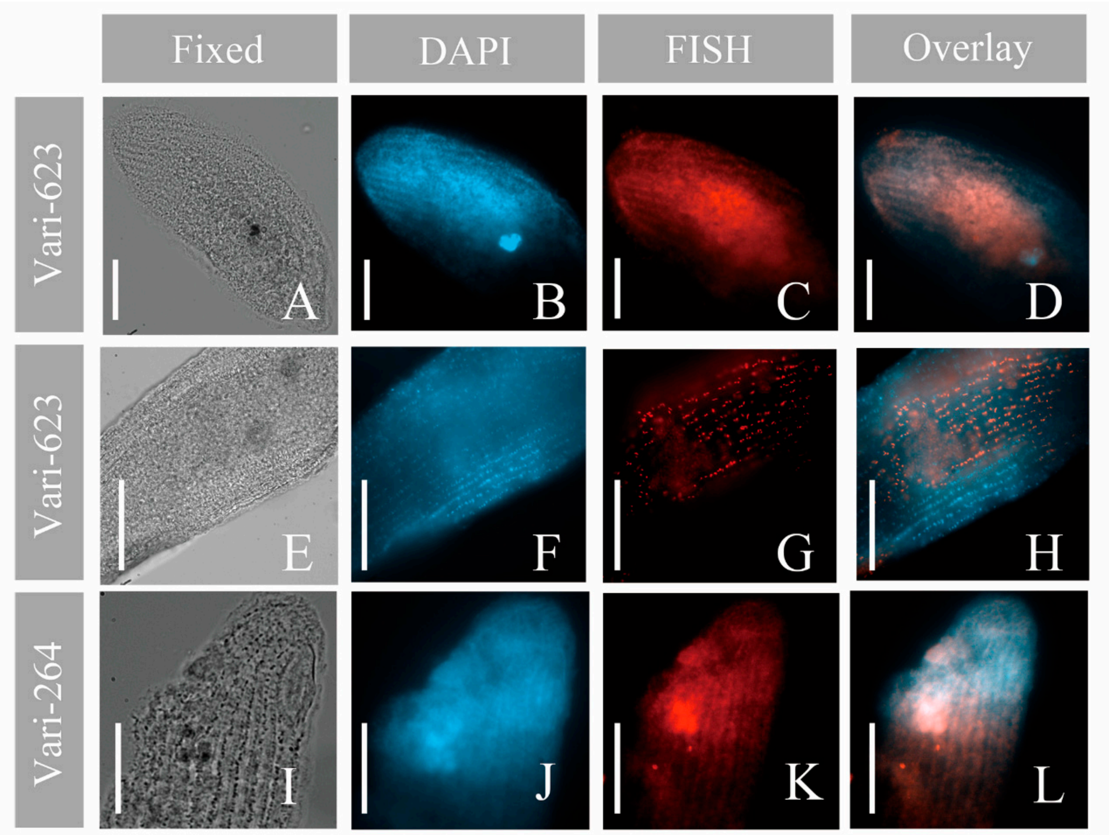

Figure S5 Micrographs of *Geleia* sp. YT with specific probes of OTU-*Variovorax* sp.. (A, E, I) Microphotographs of the fixed cells in the bright field; (B, F, J) DAPI staining; (C, G, K), and fluorescence in situ hybridization (FISH) using Vari-623 (C, G) and Vari-264 (K); (D, H, L) overlay of the DAPI and FISH. Scale bars = 10 μm (A–L). The cells Vari-623-1 and Vari-264 were collected from site A while the cell Vari-623-2 was collected from site B.

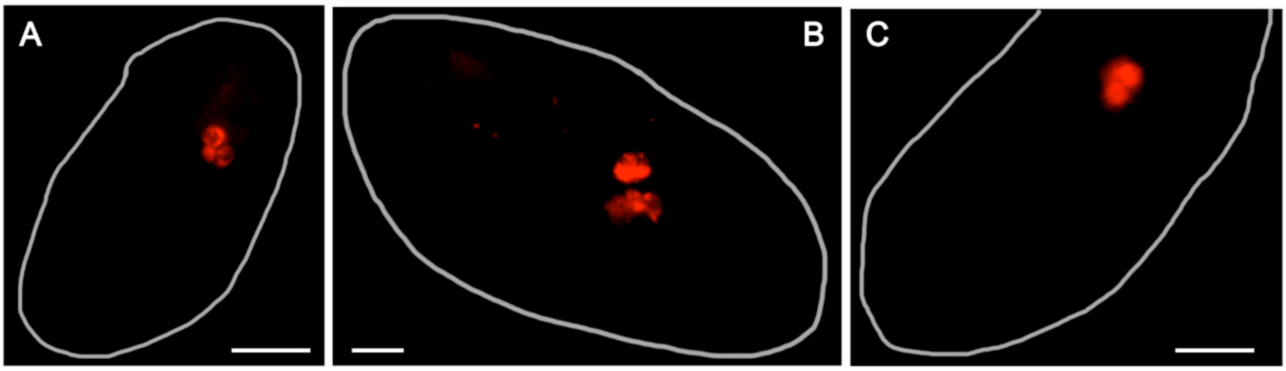

Figure S6 FISHs with Myco-692/910 probes for *Geleia* sp. YT on different sample dates. (A) Myco-692 (2022-07); (B) Myco-910 (2022-08); (C) Myco-910 (2022-09). Grey outlines represent *Geleia* cells and were drawn based on the corresponding bright field pictures. Scale bars = 10 µm (A, C); 5µm (B). The cells were collected from site A.
